# A register-based study comparing planned rehabilitation following acute stroke in 2011 and 2017

**DOI:** 10.1038/s41598-021-02337-5

**Published:** 2021-11-26

**Authors:** Malin C. Nylén, Hanna C. Persson, Tamar Abzhandadze, Katharina S. Sunnerhagen

**Affiliations:** 1 Institute of Neuroscience and Physiology, Rehabilitation Medicine, University of Gothenburg, Per Dubbsgatan 14, fl. 3, 413 45 Gothenburg, Sweden; 2 Department of Occupational Therapy and Physiotherapy, Sahlgrenska University Hospital, Gothenburg, Sweden; 3 Neurocare, Sahlgrenska University Hospital, Gothenburg, Sweden

**Keywords:** Epidemiology, Stroke

## Abstract

This cross-sectional, register-based study aimed to explore patterns of planned rehabilitation at discharge from stroke units in Sweden in 2011 and 2017 and identify explanatory variables for planned rehabilitation. Multivariable binary logistic regression was used to identify variables that could explain planned rehabilitation. There were 19,158 patients in 2011 and 16,508 patients in 2017 with stroke, included in the study. In 2011, 57% of patients were planned for some form of rehabilitation at discharge from stroke unit, which increased to 72% in 2017 (p < 0.001). Patients with impaired consciousness at admission had increased odds for planned rehabilitation (hemorrhage 2011 OR 1.43, 95% CI 1.13–1.81, 2017 OR 1.66, 95% CI 1.20–2.32), (IS 2011 OR 1.21, 95% CI 1.08–1.34, 2017 OR 1.49, 95% CI 1.28–1.75). Admission to a community hospital (hemorrhage 2011 OR 0.56, 95% CI 0.43–0.74, 2017 OR 0.39, 95% CI 0.27–0.56) (IS 2011 OR 0.63, 95% CI 0.58–0.69, 2017 OR 0.54, 95% CI 0.49–0.61) or to a specialized non-university hospital (hemorrhage 2017 OR 0.66, 95% CI 0.46–0.94), (IS 2011 OR 0.90, 95% CI 0.82–0.98, 2017 OR 0.76, 95% CI 0.68–0.84) was associated with decreased odds of receiving planned rehabilitation compared to admission to a university hospital. As a conclusion severe stroke was associated with increased odds for planned rehabilitation and patients discharged from non-university hospitals had consistently decreased odds for planned rehabilitation.

## Introduction

The global estimated disability adjusted life years (DALYs) due to stroke was more than 116 million in 2016^1^. Multidisciplinary team-based rehabilitation as well as early, supported discharge with home-based rehabilitation are effective interventions for reducing the odds of death and functional dependency after stroke^2,3^. Access to neurorehabilitation has been identified as a critical problem in acute stroke care in Europe, including Sweden^3^. In Sweden, health care including rehabilitation services, is publicly funded through taxes, and rehabilitation should be offered in proportion to individual needs. However, decisions regarding healthcare vary depending on region.

Patients with normal cognition after stroke, ability to follow instructions, and presence of a spouse or relative have been shown to be more likely to receive inpatient rehabilitation^4^. Disability or functional dependency prior to stroke, severe behavioral problems, and living far from a rehabilitation center were identified as unfavorable predictors of receiving inpatient rehabilitation^4^. The European Stroke Organization and Stroke Alliance for Europe stroke care targets for 2030 include national plans that encompass the entire chain of care, access to early rehabilitation for at least 90% of stroke survivors, and review of each patient’s rehabilitation needs at 3 months, 6 months, and annually thereafter^5^. The World Health Organization’s “Rehabilitation 2030” initiative stated that sufficient rehabilitation services are needed for an increasing population living with disability and requested that rehabilitation should be available to people regardless of their age^6^. There is a need to analyze the extent of reaching the targets for stroke rehabilitation in a country with publicly funded healthcare and to identify possible differences in factors associated with planned rehabilitation during two separate periods.

This study aimed to explore patterns of planned rehabilitation at discharge from acute stroke care in Sweden in 2011 and 2017 and identify eligible explanatory variables for planned rehabilitation.

## Materials and methods

### Study population

**Figure 1:**
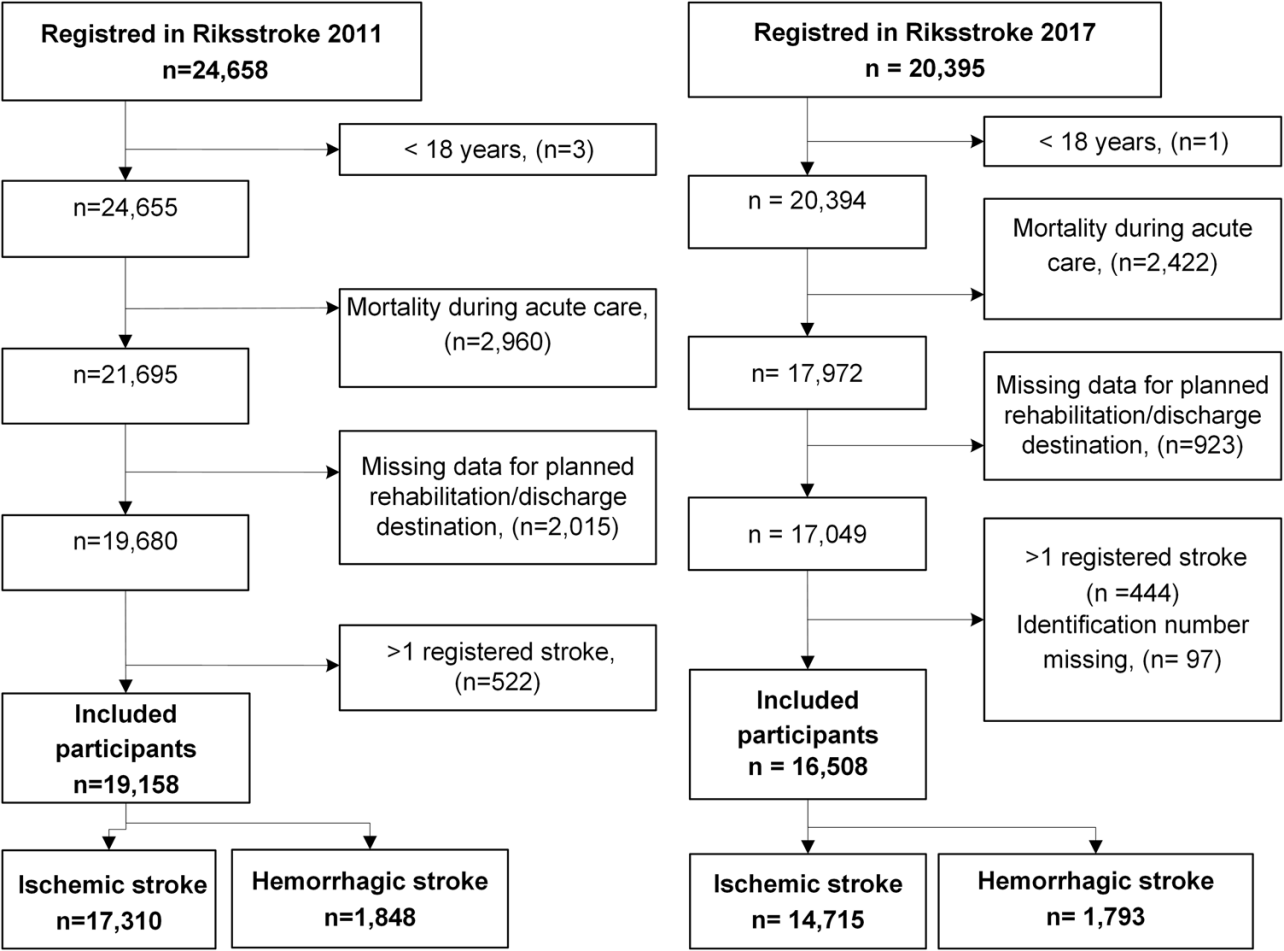
Flow chart showing the enrollment of participants in the study. Data from 2011 (left) and 2017 (right) are shown separately.

This was a cross-sectional, register-based study. Stroke population data for 2011 and 2017 were retrieved from Riksstroke, the Swedish stroke quality registry which included data from all hospitals in Sweden treating acute stroke and covered 90% of stroke cases in the country. All hospitals in Sweden with stroke care participate. The patients are treated at the closest hospital except for thrombectomy and need for neurosurgical intervention. In such cases the patient is transferred to the university hospital for intervention and then return to the local hospital. The acute care form included questions about pre-stroke health and function of daily living, stroke severity (fully conscious, drowsy, unconscious), and treatment received during hospitalization^7^. Patients 18 years or older who were diagnosed with stroke according to the International Classification of Disease-10 criteria (I61: hemorrhage, I63: cerebral infarction, and I64: unspecified cerebrovascular disease) were included in the study. Patients who died during hospitalization or missing data on both of the following: planned rehabilitation after discharge from acute stroke care and discharge destination were excluded (Fig. 1). Patients were only included once per year; in the case of multiple stroke episodes, the first episode was considered. If the next hospitalization was less than four days from previous stroke onset, it was considered as a single episode (Fig. 1).

The study complied with the Declaration of Helsinki and was approved by the Gothenburg Regional Ethical Review Board, DNR 2019-02694. Quality registers are exempt to the general rule of patient consent according to the Personal Data Act (Swedish law No. SFS 1998:204), since the information is of public interest, but there is opt out possibility.

### Variables

The variable *Planned rehabilitation* was based on registrations in the register when discharged from the stroke unit. The variable has several options. Patients were categorized as having planned rehabilitation if they were discharged to nursing homes, geriatric or rehabilitation clinics, inpatient rehabilitation, outpatient rehabilitation, day rehabilitation, or to early supported discharge. The level of rehabilitation service varies widely. At the nursing homes, occasional rehabilitation service is provided. In geriatric or rehabilitation clinics as well as inpatient rehabilitation, rehabilitation generally is provided by several professions in a team. In outpatients care, the rehabilitation is delivered usually by a single profession. Day rehabilitation on the other hand, usually is team based and delivered several times per week. In early supported discharge, the patient continues the rehabilitation with a multidisciplinary team (usually emanated from the hospital), in the patient’s home environment. Patients were categorized as *no planned rehabilitation* if the following was noted: “rehabilitation need but no access,” “patient responsible for exercise,” “no rehabilitation need,” or “no rehabilitation planned.”

*Stroke type* was categorized as ischemic stroke (IS) or hemorrhage. Patients diagnosed with I64, unspecified cerebrovascular disease were included in the IS group. At arrival, a clinical assessment as well as a CT scan is part of the routine, and an acute bleeding would most likely be detected during the examination. Thus, it is reasonable to assume these patients had an IS.

*Hospitals* were categorized according to the Riksstroke registry as university hospitals, specialized non-university hospitals, or community hospitals^8^.

*Level of consciousness on admission* was assessed using the Reaction Level Scale (RLS-85) and was used to indicate stroke severity^9^. The RLS-85 categorizes levels of consciousness as follows: alert (RLS 1), drowsy (RLS 2–3), and unconscious (RLS 4–8)^9^.

*Region* included the 21 regions in Sweden (covering all the country). The tax funded health care in Sweden is delivered in 21 independent regions, with own economic conditions. The level of rehabilitation facilities vary between regions but there are national recommendations. This variable was used to describe the patterns of planned rehabilitation.

*Reperfusion therapy* was defined as thrombolysis, thrombectomy, or both.

*Dependency in basic activities of daily living (ADL) before stroke* was defined as requiring another person’s help during the following tasks: walking indoors or outdoors, toilet activities, and/or dressing. Sociodemographic variables were also analyzed.

### Statistical analysis

Data from 2011 and 2017 as well as for IS and hemorrhage were analyzed separately. Differences between included and excluded patients as well as between groups were calculated using Pearson’s χ^2^ test for dichotomized variables and the Mann–Whitney U test for continuous variables. Data regarding planned rehabilitation or discharge destination were converted to dichotomized variables (planned rehabilitation at discharge from acute stroke care and no planned rehabilitation). Proportions for the 21 regions in total, was computed to evaluate the national mean. The national mean was compared with the proportion for each region respectively.

Binary logistic regression analyses were performed to identify eligible explanatory variables for planned rehabilitation at discharge from acute stroke care (coded as 1), which was the dependent variable. The independent variables were selected based on previous studies^8,10,11^, and were as follows: consciousness on admission, age, pre-stroke ADL dependency, previous stroke, sex, living alone, and hospital type. *Reperfusion therapy* was included as an independent variable in the IS models. Patients with missing information on any of the independent variables were excluded to enable comparison between models with identical sample sizes. Maximum likelihood estimation was performed with backward removal using the Akaike Information Criterion^12^. The final model was selected based on the lowest Akaike Information Criterion value^12^ and compared with the model containing all the variables. The Hosmer and Lemeshow test (p > 0.05 indicates good fit) and receiver operating characteristic curves^13^ were computed to check the goodness of fit. Nagelkerke R^2^ was used to show the models’ explanatory value. Odds ratios (OR), 95% confidence intervals, and p-values from binary logistic regression were presented as the results.

The level of significance was set at α = 5%. SPSS for Windows, version 25.0 (Armonk, New York, United States of America) was used for descriptive statistics. GraphPad prism (Version 9, GraphPad Software, Inc., http://www.graphpad.com) was used for figures. Binary logistic regressions were performed in R version 4.0.2 (Team RC, Vienna, Austria). The packages *Modern Applied statistics with S*^14^, and *Lasso and Elastic-Net Regularized Generalized Linear Models* (Glmnet)^15^ were used. The licenses for SPSS and GraphPad prism were provided by the University of Gothenburg. R is a free software environment that can be downloaded from https://cran.r-project.org/bin/windows/base/.

## Results

### Demographics of the participants

From 24,658 patients with stroke in 2011, 19,158 were included in the analysis. Similarly, from 20,393 patients with stroke in 2017, and 16,508 were included (Fig. 1). Excluded patients (n = 5498) had significantly higher age in 2011 (median 80.0, p < 0.001) compared to the included patients. A higher proportion of the excluded patients had impaired consciousness and hemorrhagic stroke in 2011 (p < 0.001). In 2017, there were more females (p < 0.001) and patients with unaffected consciousness (p = 0.007) among the excluded patients. Most patients were alert on admission (Table 1). The proportion of patients receiving reperfusion therapy increased significantly from 2011 to 2017. In both 2011 and 2017, patients with hemorrhage were generally younger and had lower levels of consciousness (RLS 2–8) than patients with IS. The median length of stay at the stroke unit decreased from 7 days in 2011 to 5 days in 2017. In both years, patients with hemorrhage stayed 6 days longer than patients with IS (Table 1).

**Table 1:** Characteristics of the participants.

	2011 (n = 19,158)			2017 (n = 16,508)		
	All	IS n = 17,310	Hemorrhage n = 1848	All	IS n = 14,715	Hemorrhage n = 1793
Age, median (min–max)	77 (18–103)*	78 (18–103)	75 (20–101)	76 (18–105)*	77 (18–105)	74 (18–99)
Age, mean (SD)	75.4 (12.1) n.s	75.7 (11.9)	72.6 (13.4)	74.9 (12.4) n.s	75.3 (12.2)	72.4 (13.6)
**Sex**						
Men, n (%)	9898 (51.7)*	8937 (51.6)	961 (52.0)	8851 (53.6)*	7856 (53.4)	995 (55.5)
Women, n (%)	9260 (48.3)*	8373 (48.4)	887 (48.0)	7657 (46.4)*	6859 (46.6)	798 (44.5)
**Level of consciousness, n (%)**	*Missing n* = 217			*Missing n* = 209		
Alert RLS 1	16,772 (87.5)*	15,436 (90.2)	1336 (73.3)	14,765 (89.4)*	13,378 (90.9)	1387 (77.4)
Drowsy RLS 2–3	1808 (9.4) n.s	1422 (8.3)	386 (21.2)	1290 (7.8) n.s	991 (6.7)	299 (16.7)
Unconscious RLS 4–7	361 (1.9) n.s	261 (1.5)	100 (5.5)	244 (1.5) n.s	159 (1.1)	85 (4.7)
Received thrombolysis, n (%)	1196 (6.4)* *Missing n* = 133	1178 (6.8) *Missing n* = 120	3 (0.2)	1766 (10.7)* *Missing n* = 5	1766 (12.0) *Missing n* = 5	0 (0.0)
Received thrombectomy, n (%)	80 (0.4)* *Missing n* = 133	80 (0.5) *Missing n* = 120	0 (0.0)	262 (1.6)* *Missing n* = 1842	262 (1.8) *Missing n* = 49	0 (0.0)
Living alone, n (%)	9493 (49.6)* *Missing n* = 87	8625 (50)	868 (47.3)	7760 (47.0)* *Missing n* = 100	6974 (47.4)	786 (43.8)
Previous stroke, n (%)	4388 (22.9)* *Missing n* = 117	4006 (23.3)	382 (20.8)	3421 (20.7)* *Missing n* = 29	3010 (20.5)	411 (22.9)
Atrial fibrillation Diagnosis, n (%)	5166 (27)* *Missing n* = 117	4822 (28)	344 (18.7)	3,267 (19.8)* *Missing n* = 23	2869 (19.5)	398 (22.2)
Medical treatment for hypertension, n (%)	11,603 (60.6) * *Missing n* = 92	10,612 (61.6)	991 (54.0)	10,386 (62.9)* *Missing n* = 32	9345 (63.5)	1041 (58.1)
Diabetes diagnosis, n (%)	3866 (20.2)* *Missing n* = 48	3584 (20.8)	282 (15.3)	3727 (22.6)* *Missing n* = 19	3427 (23.3)	300 (16.7)
Length of stay at hospital in total, days in median (min–max)	10 (1–100)*	10 (1–100)	19 (1–100)	8 (1–100)*	8 (1–100)	16 (1–100)
Length of stay at stroke unit, days in median (min–max)	7 (0–100)*	6 (0–100)	12 (0–100)	5 (0–100)*	5 (0–100)	11 (0–100)

*IS* ischemic stroke, *RLS* reaction level scale, *SD* standard deviation, *n.s* no significant difference between value from 2011 to 2017.

Significance levels for differences between the study populations from 2011 and 2017: *indicates p < 0.001. Pearson’s χ^2^ test (dichotomized variables) and Mann–Whitney U-test (continuous variables) were used for analysis.

### Type of rehabilitation planned for IS and hemorrhage

The proportion of patients with IS who received planned rehabilitation increased from 57 in 2011 to 71% in 2017 (p < 0.001) (Table 2). The types of rehabilitation changed from 2011 to 2017, and early supported discharge was performed more frequently in 2017. The proportion of patients with hemorrhage receiving planned rehabilitation increased from 65% in 2011 to 80% in 2017 (p < 0.001) (Table 2).

**Table 2:** Type of planned rehabilitation at discharge from a stroke unit.

	2011			2017		
	All patients n = 19,158	IS n = 17,310	Hemorrhage n = 1848	All patients n = 16,508	IS n = 14,715	Hemorrhage n = 1793
Some form of planned rehabilitation, n (%)	10,885 (56.8)*	9693 (56.8)	1192 (64.5)	11,858 (71.8)*	10,418 (70.8)	1440 (80.3)
Early supported discharge, n (%)	3405 (17.8)	3152 (18.2)	253 (13.7)	3983 (24.2)	3652 (24.8)	331 (18.5)
Outpatient rehabilitation, n (%)	1234 (6.4)	1117 (6.5)	117 (6.3)	1386 (8.4)	1270 (8.6)	116 (6.5)
Day rehabilitation, n (%)	1599 (8.4)	1442 (8.4)	157 (8.5)	1164 (7.0)	1036 (7.0)	128 (7.1)
Geriatric or rehabilitation clinic, n (%)	3095 (16.2)	2644 (15.3)	451 (24.4)	2246 (13.6)	1859 (12.6)	387 (21.6)
Inpatient rehabilitation, n (%)	476 (2.4)	413 (2.3)	63 (3.4)	319 (1.9)	278 (1.9)	41 (2.3)
Nursing home, n (%)	1214 (6.3)	1063 (6.1)	151 (8.2)	2761 (16.7)	2324 (15.8)	437 (24.4)
Rehabilitation need, no access, n (%)	NA	NA	NA	19 (0.1)	18 (0.1)	1 (0.1)
No rehabilitation planned, n (%)	8135 (42.5)	7479 (43.2)	656 (35.5)	4630 (28.0)	4296 (29.2)	353 (19.7)

*IS* ischemic stroke.

Significance levels for differences between the study populations from 2011 and 2017: *indicates p < 0.001. Pearson’s χ^2^ test was used for analysis.

### Regional deviation from mean frequency of planned rehabilitation

**Figure 2:**
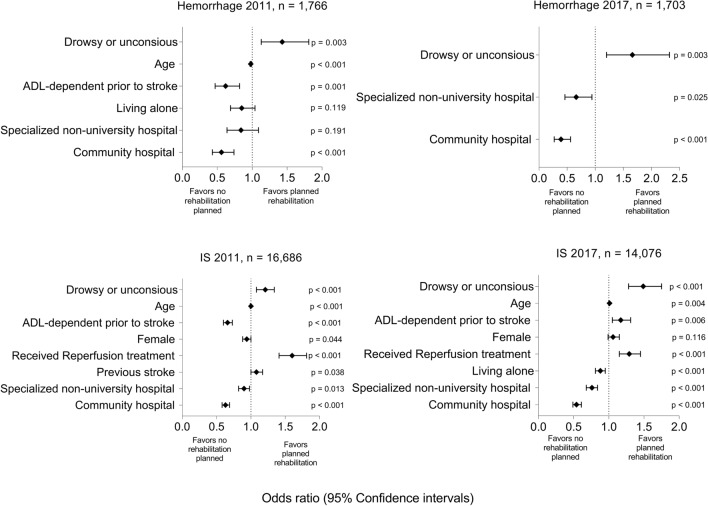
Deviation from the national mean for each of the 21 regions in Sweden regarding the percentage of patients receiving planned rehabilitation at discharge from a stroke unit. Regions with higher frequency than the national mean are shown on the positive X-axis while regions with lower frequency are shown on the negative X-axis. *Indicates regions with a change in frequency of planned rehabilitation of 30 percentage points or more between 2011 and 2017. *IS* ischemic stroke.

There were regional variations in the proportions of patients receiving planned rehabilitation at discharge from acute stroke care (Fig. 2), which were less pronounced in 2017 than in 2011 regardless of stroke type. In 2011, the range of proportions of patients receiving planned rehabilitation based on region was 19–83% for patients with IS and 29–76% for patients with hemorrhage. In 2017, the range was 47–93% for patients with IS and 59–91% for patients with hemorrhage (Fig. 2).

### Results of multivariable binary logistic regression analyses and explanatory variables for planned rehabilitation

#### Hemorrhagic stroke: 2011

**Figure 3:**
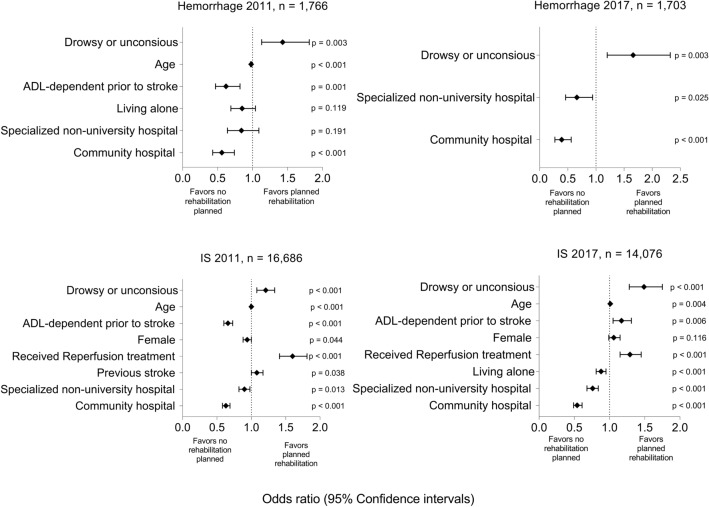
Results of the multivariable binary logistic regression analysis of planned rehabilitation presented with odds ratios and 95% confidence intervals. In 2011, 16,686 and 1766 patients with ischemic and hemorrhagic stroke were included, respectively. Hemorrhage 2011: AUC 0.63. IS 2011: AUC: 0.57. In 2017, 14,046 and 1703 patients with ischemic and hemorrhagic stroke were included, respectively. Hemorrhage 2017: AUC: 0.61. IS 2017: AUC: 0.585. *IS* ischemic stroke, *OR* odds ratio, *CI* confidence interval, *AUC* area under the ROC (receiver operating characteristics) curve.

Patients who were drowsy or unconscious on admission had higher odds of receiving planned rehabilitation than those who were alert (OR 1.43, 95% CI 1.13–1.81). Patients had lower odds of receiving planned rehabilitation if they had pre-stroke ADL dependency (OR 0.62, 95% CI 0.47–0.82, or were admitted to a community hospital (OR 0.56, 95% CI 0.43–0.74) (Fig. 3, Supplemental Table S1).

#### Ischemic stroke: 2011

Patients receiving reperfusion therapy had higher odds of receiving planned rehabilitation than those not receiving it (OR 1.60, 95% CI 1.41–1.81). Patients with pre-stroke ADL dependency had lower odds of receiving planned rehabilitation (OR 0.62, 95% CI 0.60–0.73) than those without it. Compared to patients admitted to university hospitals, patients admitted to specialized non-university hospitals and community hospitals had lower odds of receiving planned rehabilitation (OR 0.9, 95% CI 0.82–0.98 and 0.63, 95% CI 0.58–0.69, respectively; Fig. 3, Supplemental Table S1).

#### Hemorrhagic stroke: 2017

Patients who were drowsy or unconscious on admission had higher odds of receiving planned rehabilitation than those who were alert (OR 1.66, 95% CI 1.2–2.32). Compared to patients admitted to university hospitals, patients who were admitted to specialized non-university hospitals or community hospitals had lower odds of receiving planned rehabilitation (OR 0.66, 95% CI 0.46–0.94 and 0.39, 95% CI 0.27–0.56, respectively; Fig. 3, Supplemental Table S2).

#### Ischemic stroke: 2017

Patients had higher odds of receiving planned rehabilitation if they had reperfusion therapy (OR 1.29, 95% CI 1.15–1.45) and pre-stroke ADL dependency (OR 1.17, 95% CI 1.05–1.31). Admission to a specialized non-university hospital (OR 0.76, 95% CI 0.68–0.84) or community hospital (OR 0.54, 95% CI 0.49–0.61) were associated with lower odds of receiving planned rehabilitation compared to admission to a university hospital (Fig. 3, Supplemental Table S2).

## Discussion

The results of this register-based study showed that regardless of stroke type, the proportion of patients receiving planned rehabilitation at discharge from a stroke unit increased from 2011 to 2017. Regional variation was observed in both years. Explanatory variables for planned rehabilitation were different depending on year and stroke type. Patients with impaired consciousness on arrival at the hospital had increased odds for planned rehabilitation regardless of year and stroke type. Admission to non-university hospitals was consistently associated with decreased odds of receiving planned rehabilitation.

The proportion of patients receiving planned rehabilitation at discharge from a stroke unit increased significantly from 2011 to 2017. Within this time period, two revisions of National stroke guidelines have been published. This may lead to increased awareness of the patient’s rehabilitation needs. The median length of stroke unit stay was 2 days shorter in 2017 than in 2011, and it is possible that earlier discharge corresponds with more frequent referral for rehabilitation. Development of rehabilitation facilities such as ESD, could also contribute to increased detection of rehabilitation needs. ESD has been shown to reduce the odds of death or dependency^3^. According to Swedish national stroke care guidelines, ESD should be offered to patients with mild to moderate stroke^16^. This knowledge has grown over the years, and has in the revisions of national guidelines given a higher priority each time. In this study, the proportion of patients who received ESD increased from 2011 to 2017 in line with the guidelines. This can be taken as an indicator for successful implementation of the guidelines in clinical praxis. The content and intensity of other types of rehabilitation interventions, remains unknown.

Patients treated at specialized non-university hospitals or community hospitals had consistently lower odds of receiving planned rehabilitation compared to those treated at university hospitals. There are several possible explanations for this. First, non-university hospitals are often located in smaller towns with a larger geographical coverage than university hospitals; thus, it is possible that patients lived far from rehabilitation centers. Long distances between patients’ homes and rehabilitation centers have previously been associated with decreased probability of referral to rehabilitation^4^. Second, there may have been fewer rehabilitation facilities available in these areas. Third, university hospitals are also education centers, and may be staffed by professionals that smaller hospitals are unable to employ.

Patients with IS with pre-stroke ADL dependency and those who were older were less likely to receive planned rehabilitation in 2011. In contrast, higher age and ADL dependency were favorably associated with planned rehabilitation in patients with IS in 2017. Post-stroke referral patterns in Canada showed that younger and more functionally impaired patients were referred for inpatient and outpatient rehabilitation more often than older or functionally independent patients^17,18^. ADL dependency prior to stroke and older age have been shown to be unfavorably associated with rehabilitation access^19^. The results from this study indicate an increased awareness of rehabilitation needs for older adults and individuals with dependency in basic ADL. This may be due to the ongoing quality assurance in Swedish healthcare that aim to supply individualized rehabilitation to support healthiness in different circumstances of life.

Regional variation was observed in both 2011 and 2017 and are unlikely to be completely explained by differences in patient characteristics. Inequalities in access to rehabilitation based on geographical location, is unaccepted given that equal conditions to achieve health is stated in the Swedish law. Local differences in referral protocols could have contributed to the observed variation. In addition, there is no standardized tool to assess rehabilitation need after stroke in general. Taken altogether, there is a need to implement a strategy that ensure that the actual rehabilitation is planned and delivered to the patient according to the rehabilitation needs.

### Strengths and limitations

The current study was based on a large dataset from the Riksstroke registry. Tax funded health care gives the opportunity for each person to receive care. The present study had a few limitations. Register-based data commonly includes missing data, due to administrative issues, causing patient dropout. Furthermore, the types of rehabilitation eligible in the registry changed between 2011 and 2017 and data regarding rehabilitation and discharge destination was dichotomized, causing reduced generalizability to clinical practice. In the register, data regarding the patients’ National Institutes of Health Stroke Scale scores were missing. As a proxy, the consciousness on admission, assessed using the RLS-85^9^, was used to evaluate stroke severity. The RLS-85 is a part of the National Institutes of Health Stroke Scale, and consciousness level is considered as a reliable predictor of post-stroke survival^9^. Socioeconomic factors such as income, education level, and country of birth were not available from the Riksstroke registry, and these factors could possibly affect planned rehabilitation. Lastly, the present study is based on cohorts from 2011 and 2017, and if continuous data had been available between the years, perhaps a better understanding on patterns of planned rehabilitation could have been obtained.

## Conclusions

Regardless of stroke type, the proportion of patients with planned rehabilitation from stroke units increased from 2011 to 2017. There were pronounced regional variations regarding patients with planned rehabilitation both years. Severe stroke was associated with increased odds for planned rehabilitation. Patients discharged from non-university hospitals had consistently decreased odds for planned rehabilitation. The increased proportion with planned rehabilitation in 2017 could indicate improved awareness for rehabilitation needs. The regional variations and the impact of hospital type though disclose a need for future development in line with the European Stroke Organization’s targets for 2030 and the World Health Organization’s “Rehabilitation 2030: a call for action”.

## Supplementary Information

Supplementary Tables.

## Data Availability

Due to the sensitive nature of the data collected for this study, open access cannot be provided. Requests to access the dataset can be submitted from qualified researchers to the authors (contact Professor Katharina S. Sunnerhagen, email: ks.sunnerhagen@neuro.gu.se). According to the Swedish regulation http://www.epn.se/en/start/regulations/, data can only be used in accordance with the application for this study that was approved by the Ethical board.
