# Correction: A register-based study comparing planned rehabilitation following acute stroke in 2011 and 2017

**DOI:** 10.1038/s41598-026-46029-4

**Published:** 2026-04-21

**Authors:** Malin C. Nylén, Hanna C. Persson, Tamar Abzhandadze, Katharina S. Sunnerhagen

**Affiliations:** 1 Institute of Neuroscience and Physiology, Rehabilitation Medicine, University of Gothenburg, Per Dubbsgatan 14, Fl. 3, 413 45 Gothenburg, Sweden; 2 Department of Occupational Therapy and Physiotherapy, Sahlgrenska University Hospital, Gothenburg, Sweden; 3 Neurocare, Sahlgrenska University Hospital, Gothenburg, Sweden

Correction to: *Scientific Reports* 10.1038/s41598-021-02337-5, published online 26 November 2021

The original version of this Article contained an error. Figure 2 in the original version of this article was a duplicate of Figure 3.

The correct Figure 2 along with the caption appear below:

**Fig. 2:**
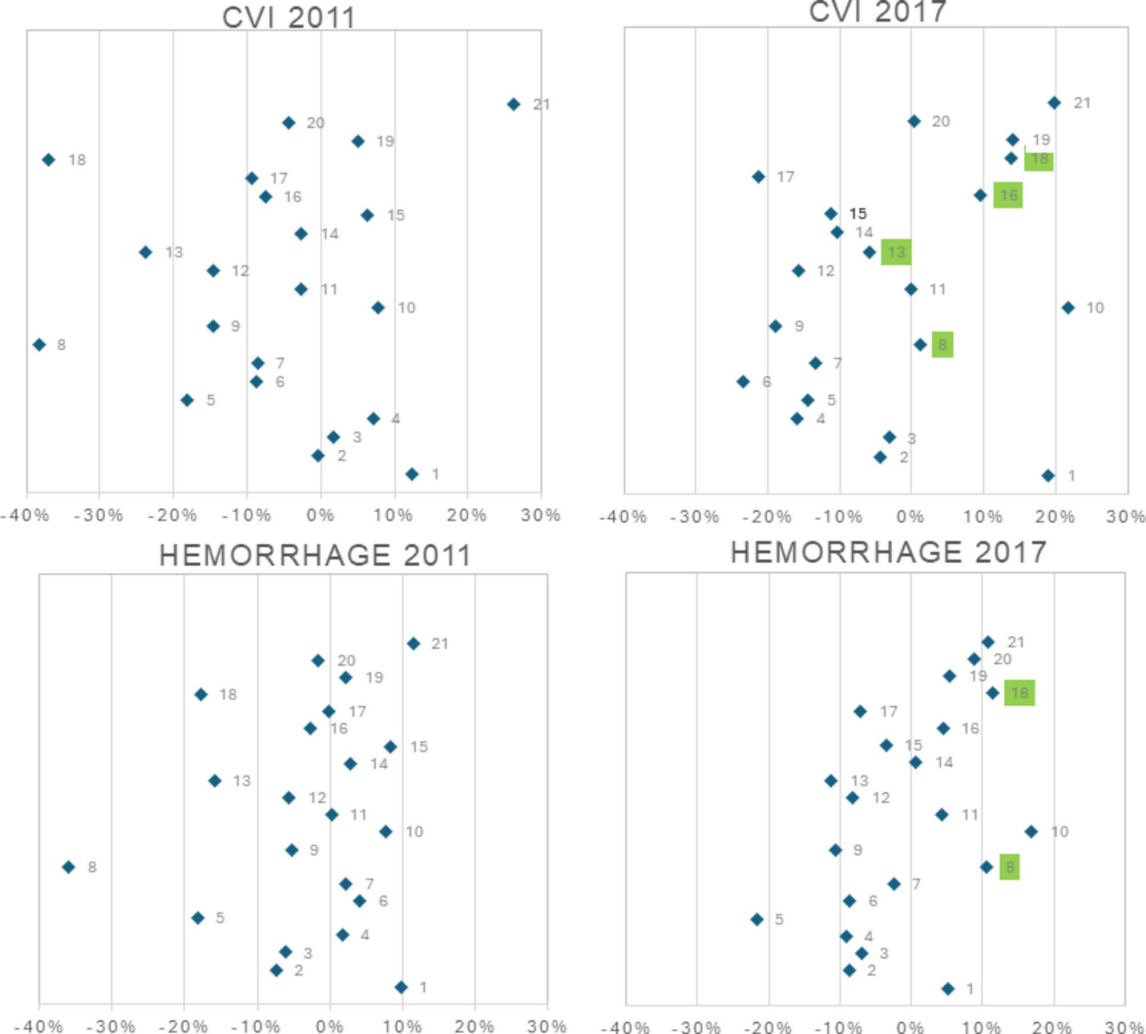
Deviation from the national mean for each of the 21 regions in Sweden regarding the percentage of patients receiving planned rehabilitation at discharge from a stroke unit. Regions with higher frequency than the national mean are shown on the positive X-axis while regions with lower frequency are shown on the negative X-axis. *Indicates regions with a change in frequency of planned rehabilitation of 30 percentage points or more between 2011 and 2017. IS ischemic stroke.

