# Supplementary material for: A register-based study comparing planned rehabilitation following acute stroke in 2011 and 2017
Source: Sci Rep. 2021 Nov 26;11:23001. doi: 10.1038/s41598-021-02337-5 (PMC8626515; doi:10.1038/s41598-021-02337-5)
Supplement: Supplementary file 1 — Supplementary Tables. [file 41598_2021_2337_MOESM1_ESM.docx]

**Supplemental material**

## **Title:** A register-based study comparing planned rehabilitation following acute stroke in 2011 and 2017

**Authors:** Malin Nylén MSc^1^, Hanna C. Persson PhD^1^, Tamar Abzhandadze MSc^1, 2^,
Katharina S. Sunnerhagen MD, PhD^1^,
^1^University of Gothenburg, Institute of Neuroscience and Physiology, Rehabilitation medicine, University of Gothenburg. Gothenburg, Sweden. Per Dubbsgatan 14, fl. 3, 413 45 Gothenburg, Sweden

^2^Department of Occupational Therapy and Physiotherapy, Sahlgrenska University Hospital, Gothenburg, Sweden

**Supplemental Tables**

Supplemental table S1. Results of multivariable binary logistic regression for planned rehabilitation in 2011 including patients with ischemic (n=16,686) and hemorrhagic (n=1,766) stroke

| Hemorrhage, n = 1,766 | | | |
| --- | --- | --- | --- |
|  | Adjusted p-value | Adjusted OR | Adjusted 95 % CI for OR |
| RLS 2–8  Reference: RLS 1 | 0.003 | 1.43 | 1.13–1.81 |
| Age, change per year | <0.001 | 0.98 | 0.97–0.99 |
| Living alone  Reference: Living together with someone | 0.119 | 0.85 | 0.69–1.04 |
| Pre-stroke ADL dependency  Reference: Pre-stroke ADL independence | 0.001 | 0.62 | 0.47–0.82 |
| Hospital type  Reference: University Hospital | <0.001 |  |  |
| Specialized non-university hospital | 0.191 | 0.84 | 0.64–1.09 |
| Community hospital | <0. 001 | 0.56 | 0.43–0.74 |
| IS, n = 16,686 | | | |
|  | Adjusted p-value | Adjusted OR | Adjusted 95 % CI for OR |
| RLS 2–8  Reference: RLS 1 | <0. 001 | 1.21 | 1.08–1.34 |
| Pre-stroke ADL dependency  Reference: Pre-stroke ADL independence | <0. 001 | 0.66 | 0.60–0.73 |
| Age | <0. 001 | 1.00 | 0.99–1.00 |
| Previous stroke  Reference: First stroke | 0.038 * | 1.08 | 1.00–1.17 |
| Sex: Female  Reference: Male | 0.044* | 0.94 | 0.88–1.00 |
| Received reperfusion treatment  Reference: No reperfusion treatment | <0. 001 | 1.60 | 1.41–1.81 |
| Hospital type  Reference: University Hospital | <0. 001 |  |  |
| Specialized non-university hospital | 0.013 * | 0.90 | 0.82–0.98 |
| Community hospital | <0. 001 | 0.63 | 0.58–0.69 |
| Hemorrhage: Hosmer and Lemeshow: p = 0.55, Nagelkerke R^2^: 0.07, Area under receiver operating characteristic curve: 0.63.  IS: Hosmer and Lemeshow: p =0.04, Nagelkerke R^2^: 0.03, Area under receiver operating characteristic curve: 0.57.  Abbreviations: IS: Ischemic stroke, OR: Odds-ratio, CI: confidence interval, RLS: Reaction Level Scale, ADL: activities of daily living. | | | |

Supplemental table S2. Results of multivariable binary logistic regression for planned rehabilitation in 2017 including patients with ischemic (n=14,076) and hemorrhagic (n=1,703) stroke.

| Hemorrhage n = 1,703 | | | |
| --- | --- | --- | --- |
|  | Adjusted p-value | Adjusted OR | Adjusted 95 % CI for OR |
| RLS 2–8  Reference: RLS 1 | 0.003 | 1.66 | 1.20–2.32 |
| Hospital type  Reference: University Hospital | <0. 001 |  |  |
| Specialized non-university hospital | 0.025 | 0.66 | 0.46–0.94 |
| Community hospital | <0. 001 | 0.39 | 0.27–0.56 |
| IS, n =14 076 | | | |
|  | Adjusted p-value | Adjusted OR | Adjusted 95 % CI for OR |
| RLS 2–8  Reference: RLS 1 | <0. 001 | 1.49 | 1.28–1.75 |
| Age | 0.004 | 1.01 | 1.00–1.01 |
| Sex: Female  Reference: Male | 0.116 | 1.06 | 0.99–1.15 |
| Living alone  Reference: Living together with someone | <0. 001 | 0.88 | 0.81–0.95 |
| Pre-stroke ADL dependency  Reference: Pre-stroke ADL independence | 0.006 ** | 1.17 | 1.05–1.31 |
| Received reperfusion treatment  Reference: No reperfusion treatment | <0. 001 | 1.29 | 1.15–1.45 |
| Hospital type  Reference: University Hospital | <0. 001 |  |  |
| Specialized non-university hospital | <0. 001 | 0.76 | 0.68–0.84 |
| Community hospital | <0. 001 | 0.54 | 0.49–0.61 |
| Hemorrhage: Hosmer and Lemeshow: p =0.344, Nagelkerke R^2^: 0.039, Area under receiver operating characteristic curve: 0.611. IS: Hosmer and Lemeshow: p =0.180, Nagelkerke R^2^: 0.025, Area under receiver operating characteristic curve: 0.585. Abbreviations: IS: ischemic stroke, OR: Odds-ratio, CI: confidence interval, RLS: Reaction Level Scale, ADL: activities of daily living. | | | |
